# Panaxatriol Saponins Attenuated Oxygen-Glucose Deprivation Injury in PC12 Cells via Activation of PI3K/Akt and Nrf2 Signaling Pathway

**DOI:** 10.1155/2014/978034

**Published:** 2014-05-11

**Authors:** Yongliang Huang, Jie Yu, Fang Wan, Wenwu Zhang, Huarong Yang, Li Wang, Hongyi Qi, Chunjie Wu

**Affiliations:** ^1^College of Pharmacy, Chengdu University of Traditional Chinese Medicine, 1166 Liutai Road, Wenjiang District, Chengdu, Sichuan 611137, China; ^2^College of Pharmaceutical Sciences, Southwest University, 2 Tiansheng Road, Beibei District, Chongqing 400716, China; ^3^Huasun Group Co., Ltd., Chengdu, Sichuan 610072, China

## Abstract

Panaxatriol saponins (PTS), the main components extracted from *Panax notoginseng*, have been shown to be efficacious in the prevention and treatment of cerebrovascular diseases in China. NF-E2-related factor 2 (Nrf2), a transcription factor regulating antioxidant and cytoprotective responses to oxidative stress, has received particular attention as a molecular target for pharmacological intervention of ischemic diseases. The aim of this study was to characterize the effect of PTS on the activation of Nrf2 signaling pathway and the potential role in its protective effect. We found that PTS induced heme oxygenase-1 (HO-1) expression in PC12 cells via activating Nrf2 signaling pathway. Phosphatidylinositol 3-kinase (PI3K)/Akt kinase was involved in the upstream of this PTS activated pathway. Moreover, combination of the main components in PTS significantly enhanced the expression of Nrf2 mediated phase II enzymes. Importantly, the protective effect of PTS against oxygen-glucose deprivation-reperfusion (OGD-Rep) induced cell death was significantly attenuated by PI3K inhibitor and antioxidant response element (ARE) decoy oligonucleotides, suggesting that both PI3K/Akt and Nrf2 signaling pathway are essential during this protective process. Taken together, our results suggest that PTS may activate endogenous cytoprotective mechanism against OGD-Rep induced oxidative injury via the activation of PI3K/Akt and Nrf2 signaling pathway.

## 1. Introduction

Cerebrovascular disease has become the third in China and the fourth in America leading cause of death among adults [1, 2]. At present, the recombinant tissue plasminogen activator (rtPA) remains the only FDA-approved therapeutic agent for the treatment of ischemic stroke. However, the short therapeutic window, low rates of recanalization, only modest benefits, and high rates of mortality have limited its use [3]. There is now increasing evidence to suggest that oxidative stress plays a critical role in the pathogenesis of ischemic stroke [4]. Oxidative stress resulting from generation of reactive oxygen species (ROS) has been shown to cause neuronal damage via the opening of potassium channels and altered vascular reactivity, breakdown of the blood brain barrier, and focal destructive lesions in animal models of ischemic stroke [4, 5]. Therefore, control of the intracellular redox status may represent an alternative treatment to neurological damage caused by stroke-associated oxidative stress.

Recently, the transcription factor NF-E2-related factor 2 (Nrf2) has received particular attention as it is in the center of a redox homeostatic gene regulatory network, which will quickly and sufficiently enhance the cell's antioxidant capacity to conquer the oxidative stress [6]. Nrf2 plays a critical role in regulating the antioxidant responsive element (ARE), a key regulatory element in the promoter region of genes encoding cellular antioxidative and cytoprotective phase II enzymes including heme oxygenase-1 (HO-1) and NAD(P)H:quinone oxidoreductase 1 (NQO1) [7]. As a result, the Nrf2 pathway is suggested as a molecular target for pharmacological intervention of ischemic diseases [8].

*Panax notoginseng*, the root of* Panax notoginseng* (Burk.) F.H. Chen, has been traditionally used in China for more than 600 years as either food or herbal medicine. It is believed that* Panax notoginseng* is beneficial to the prevention and treatment of various diseases, such as cardio- and cerebrovascular diseases, diabetes, pains, and bleeding [9]. Panaxatriol saponins (PTS), one of the major effective components of* Panax notoginseng*, have been clinically used in China for the treatment of cerebral infarction [10]. Previous study showed that PTS protected against focal cerebral ischemia through reducing cerebral edema, upregulating heat shock protein HSP70 expression, and downregulating transferring [11]. A recent report indicated that pretreatment with PTS ameliorated ischemia reperfusion induced myocardial damage via reducing oxidative stress [12]. As mentioned above, the indirect antioxidative effect mediated by Nrf2 signaling pathway is beneficial for the ischemic diseases. In the present study, we designed experiments to investigate the effect of PTS on Nrf2 signaling pathway and determine its critical role in oxygen-glucose deprivation and reperfusion (OGD-Rep) induced injury.

## 2. Materials and Methods

### 2.1. Materials

**Figure 1:**
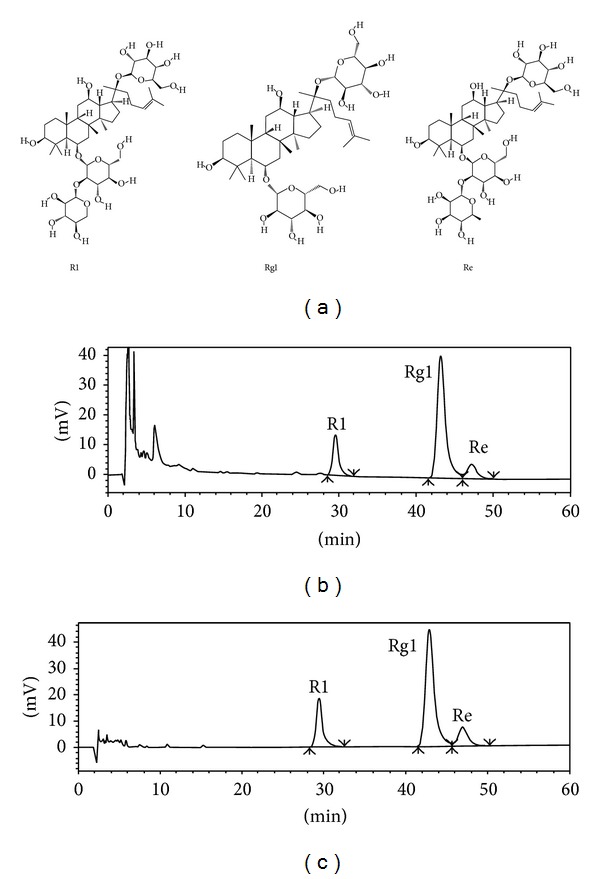
Chemical structures and representative HPLC chromatograms of PTS. (a) Chemical structures of R1, Rg1, and Re. (b) HPLC chromatogram of PTS extract. (c) HPLC chromatogram of reference compounds. UV absorbance of the HPLC samples was monitored at 210 nm.

PTS extracted from* Panax notoginseng* was obtained from Huasun Group Co., Ltd. (Sichuan, China). As determined by HPLC in Figure 1, ginsenoside Rg1 (Rg1), notoginsenoside R1 (R1), and ginsenoside Re (Re) are the main components of PTS with the concentrations of 46.1%, 12.3%, and 5.7%, respectively. HO-1 antibody was purchased from Enzo Life Sciences (NY, USA). The antibodies against Nrf2 and lamin b1 were purchased from Santa Cruz Biotechnology (CA, USA). The antibodies against *β*-actin and rabbit IgG were purchased from Sigma-Aldrich (St. Louis, MO, USA). The antibody against phospho-Akt was purchased from Cell Signaling Technology (Boston, MA, USA). Other chemicals were obtained from Sigma-Aldrich Co. (St. Louis, MO, USA) unless indicated otherwise.

### 2.2. Cell Culture

Rat pheochromocytoma PC12 cells were obtained from the American Type Cell Culture Collection (Manassas, VA) and maintained in Dulbecco's modified Eagle's medium (DMEM) supplemented with 10% horse serum (Invitrogen, USA), 5% fetal bovine serum (FBS) (Invitrogen, USA), and 1% penicillin/streptomycin (Invitrogen, USA) on collagen I coated dishes at 37°C in a humidified 5% CO_2_ atmosphere.

### 2.3. Measurement of Cell Viability

Cell viability was evaluated by a Cell Counting Kit-8 (CCK-8) assay (Dojindo Laboratories, Kumamoto, Japan), which is based on the conversion of a water-soluble tetrazolium salt, 2-(2-methoxy-4-nitrophenyl)-3-(4-nitrophenyl)-5-(2,4-disulfophenyl)-2H-tetrazolium, monosodium salt (WST-8), to a water-soluble formazan dye upon reduction by dehydrogenases in the presence of an electron carrier [13]. Briefly, at the end of drug treatment, CCK-8 solution (10 *μ*L) was added to each well, followed by incubation for 3 h at 37°C. The absorbance at 450 nm was determined by a microplate reader (Lambda Bio-20, Beckman). Cell viability was expressed as a percentage of that of the control (untreated) cells.

### 2.4. Procedure of Oxygen-Glucose Deprivation and Reperfusion (OGD-Rep)

The* in vitro* ischemia-reperfusion model was set up by OGD-Rep treatment of PC12 cells as described previously [14]. Briefly, PC12 cells were first incubated in glucose-free DMEM and subsequently transferred into a Tri-Gas incubator (Heal Force, HF100) with 1% O_2_, 94% N_2_, and 5% CO_2_ for 8 h at 37°C. Sham OGD cultures were maintained in a normal oxygenated DMEM. Following the OGD treatment, cells were returned to the normoxic incubator with normal culture medium and incubated for another 24 h.

### 2.5. Preparation of Total Cellular, Cytosolic, and Nuclear Proteins

At the end of drug treatment, the cellular proteins were extracted from cultured cells in ice-cold RIPA buffer (Cell Signaling Technologies, USA) supplemented with 1% (v/v) protein inhibitor cocktail (Sigma-Aldrich, USA) and 1 mM phenylmethylsulfonyl fluoride (PMSF). Cytosolic and nuclear extracts were isolated as described previously [14, 15]. Briefly, following 30 min incubation in ice-cold buffer A (pH 8.0) containing 20 mM* N*-2-hydroxyethylpiperazine-*N*′-2-ethanesulfonic acid (HEPES), 1 mM ethylenediaminetetraacetic acid (EDTA), 1.5 mM MgCl_2_, 10 mM KCl, 1 mM DTT, 1 mM sodium orthovanadate, 1 mM NaF, 1 mM PMSF, and 1% (v/v) protein inhibitor cocktail, the cells were lysed by adding the appropriate volume of 10% (v/v) NP-40 to a final concentration of 0.625% (v/v) and vortexing. The cell lysates were separated into the supernatant as cytosolic extract and the nuclear pellet by centrifugation. The nuclear proteins were extracted from the pellet in ice-cold buffer C (pH 8.0) containing 20 mM HEPES, 1 mM EDTA, 1.5 mM MgCl_2_, 10 mM KCl, 1 mM DTT, 1 mM sodium orthovanadate, 1 mM NaF, 1 mM PMSF, 1% (v/v) protein inhibitor cocktail, and 20% (v/v) glycerol. The concentration of the cellular proteins was determined by Protein Assay Dye Reagent Concentrate (Bio-Rad Laboratories Inc., USA) using bovine serum albumin as a standard.

### 2.6. Western Blotting Analysis

The cellular proteins were extracted and analyzed for protein expression as previously described [14]. Briefly, thirty micrograms of the cellular proteins were resolved by electrophoresis in 10% SDS-polyacrylamide gel and subsequently transferred to polyvinylidene difluoride (PVDF) membrane. Following 1 h incubation in a fresh TBS buffer containing 0.1% Tween-20 and 5% BSA, the blots were probed with specific antibodies including anti-HO-1, anti-NQO1, anti-*β*-actin, anti-phospho-Akt, anti-Nrf2, or anti-lamin b1. The bound primary antibodies were detected by horseradish peroxidase conjugated anti-rabbit IgG or anti-goat IgG accordingly. The activity of peroxidase in the blot was visualized by enhanced chemiluminescence (ECL) detection reagents (GE Healthcare, Sweden).

### 2.7. Immunostaining of Nrf2 in Cultured Cells

The nuclear translocation of Nrf2 was determined by immunostaining as previously described [14, 16]. The monoclonal antibody recognizing Nrf2 was used. Cells were cultured on collagen I coated-glass coverslips in complete growth medium. After treatment, cells were washed three times with PBS and fixed with paraformaldehyde (3.7%). After the removal of excessive paraformaldehyde, fixed cells were incubated in a fresh blocking buffer (0.5% Triton X-100 in PBS, pH 7.4, containing 10% normal goat serum) for 1 h at room temperature. Cells were then incubated overnight at 4°C on addition of anti-Nrf2 primary antibody solution (diluted 1 : 100 in PBS with 3% bovine serum albumin). The bound antibody was detected by FITC-goat anti-rabbit IgG secondary antibody (1 : 1,000) in PBS containing 3% bovine serum albumin. The cell nucleus was stained with 4′,6-diamidino-2-phenylindole (DAPI) (Invitrogen, USA). Images were captured on a Zeiss fluorescence microscope (Carl Zeiss, Germany).

### 2.8. Decoy Design and Treatment

ARE decoy oligonucleotide (ODN) was used in this study to inhibit Nrf2 mediated gene expression according to previous study [17]. Upper-strand and reverse-complement phosphorothioated ODNs were commercially synthesized and purified by Sangon Biotech Inc. (Shanghai, China). Double-stranded decoy ODNs were prepared by annealing complimentary single strands in sterile saline. The sequences are shown in Table 1 and underlining indicates the ARE core binding sequence. In addition to the ARE decoy ODNs, a scrambled decoy ODN (*mut* ODN) was used as control for specificity. To determine the transfection rate and cellular localization of ARE decoy ODNs, ODNs were 5′-end-labeled with fluorescein isothiocyanate (FITC). To increase the delivery of ODNs into the cell, lipofectamine 2000 (Invitrogen, USA) was used in the transfection treatment. The ARE decoy and ARE* mut* ODNs were added to the cells at 100 nM in the presence of lipofectamine 2000. After 24 h of incubation, PTS were added directly to the medium as described before.

### 2.9. Statistical Analysis

All data were presented as mean ± SD from three independent experiments. Statistical analysis was performed by two-tail Student's *t*-test. A *P* value of less than 0.05 was considered to be statistically significant.

## 3. Results

### 3.1. Protective Effect of PTS and Combination of Rg1, Re, and R1 against OGD-Rep Induced Injury in PC12 Cells

**Figure 2:**
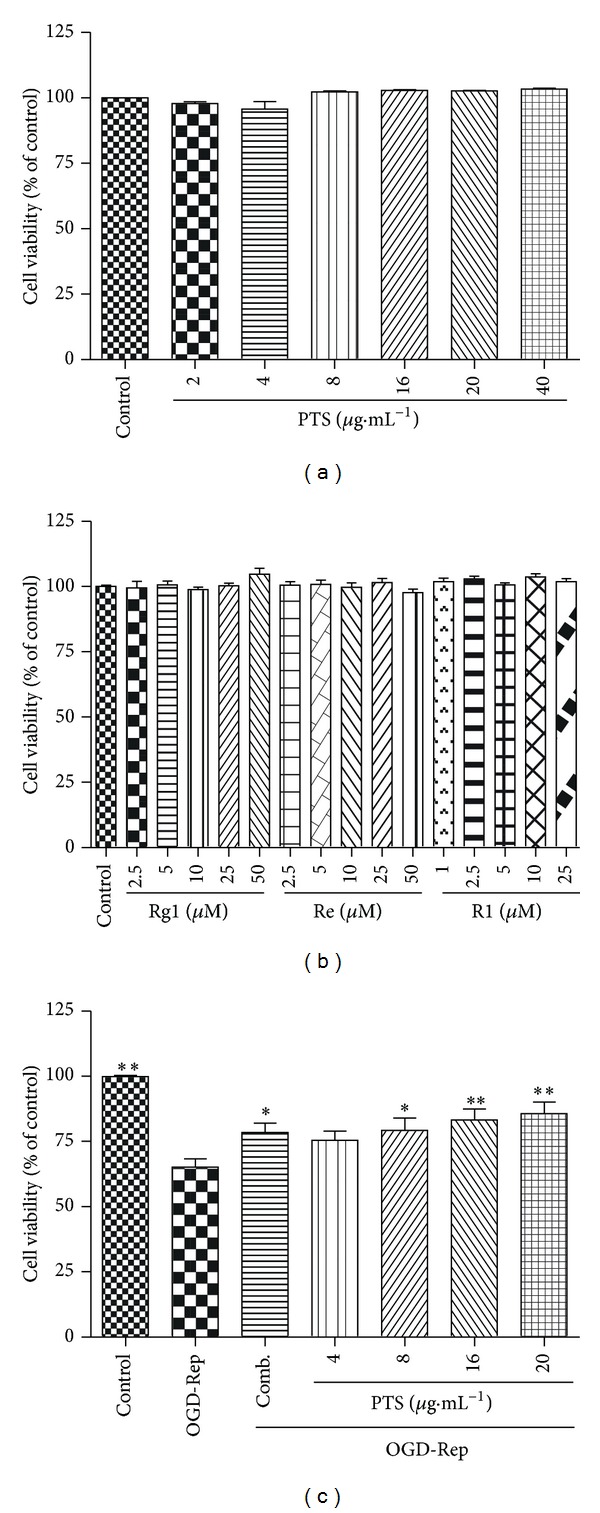
Cytotoxicity and protective effect of PTS, Rg1, Re, and R1 in PC12 cells. (a) Cytotoxicity of PTS. PC12 cells were treated with PTS at concentrations ranging from 0 to 40 *μ*g·mL^−1^ for 24 h. (b) Cytotoxicity of Rg1, Re, and R1. The treatment was the same as described in Panel A except with the indicated concentrations. (c) Effect of PTS on the cell survival of PC12 cells against OGD-Rep. PC12 cells were pretreated with PTS at concentrations ranging from 0 to 20 *μ*g·mL^−1^ or the combination of Rg1, Re, and R1 (Comb.) with each 2.5 *μ*M for 2 h, subsequently subjected to OGD-Rep treatment for 8 h, and finally maintained in oxygenated cell culture medium for another 24 h. The cell viability was determined by CCK-8 assay. Values represent mean ± SD (*n* = 6). *P < 0.05 and **P < 0.01 versus OGD-Rep.

To examine the effect of PTS and its main components Rg1, Re, and R1 at different concentrations on cellular viability, PC12 cells were treated with the drug for 24 h. The cell viability was determined using a CCK-8 assay. As shown in Figures 2(a) and 2(b), PTS did not show toxicity up to the concentration of 40 *μ*g·mL^−1^, while Rg1, Re, and Re were not toxic at experimental concentrations (0 to 50 *μ*M for Rg1 and Re, 0 to 25 *μ*M for R1, resp.).

We further addressed the question of whether PTS and the combination of its main components could protect PC12 cells against ischemia-reperfusion injury. We adopted an* in vitro* OGD-Rep procedure to mimic ischemic stroke as described [18]. As shown in Figure 2(c), OGD-Rep induced significant cell injury as indicated by CCK-8 assay. 40% of PC12 cells were killed by OGD for 8 h and reperfusion for 24 h relative to a sham control. Notably, PTS at concentrations ranging from 8 to 20 *μ*g·mL^−1^ or the combination of Rg1, Re, and R1 with each 2.5 *μ*M could significantly protect cells from OGD-Rep induced cell death. Within the concentrations tested, PTS showed a concentration-dependent response against OGD-induced injury in PC12 cells.

### 3.2. PTS Induces HO-1 Expression via Activating Nrf2 Pathway

**Figure 3:**
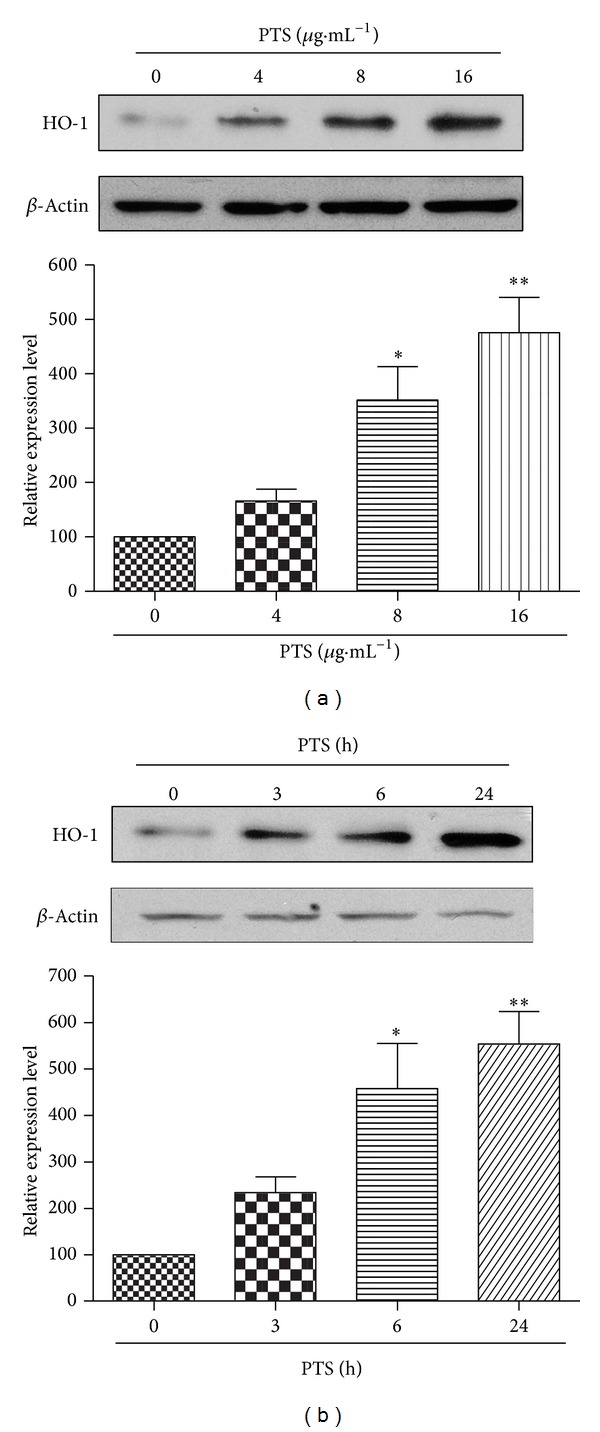
Effect of PTS on HO-1 expression. (a) Dose dependent induction of HO-1 by PTS. PC12 cells were treated with PTS at various concentrations (0–16 *μ*g·mL^−1^) for 24 h. (b) Time-dependent induction of HO-1 by PTS. PC12 cells were treated with PTS at fixed concentration (16 *μ*g·mL^−1^) for different time points as indicated. HO-1 protein was detected by Western blotting using specific antibodies, whereas *β*-actin was detected as the control. *P < 0.05, **P < 0.01, significantly different from vehicle group. The blots were representative of three independent experiments.

To determine the potential effect of PTS on Nrf2 signaling pathway, we first conducted Western blotting to characterize the inducing effect of PTS on HO-1, a typical antioxidant downstream target protein of Nrf2. As a result, HO-1 induction was positively correlated with the concentration of PTS over the range of 0 to 16 *μ*g·mL^−1^ (Figure 3(a)). Our results also showed that PTS at a fixed concentration (16 *μ*g·mL^−1^) induced HO-1 in a time-dependent manner (Figure 3(b)).

**Figure 4:**
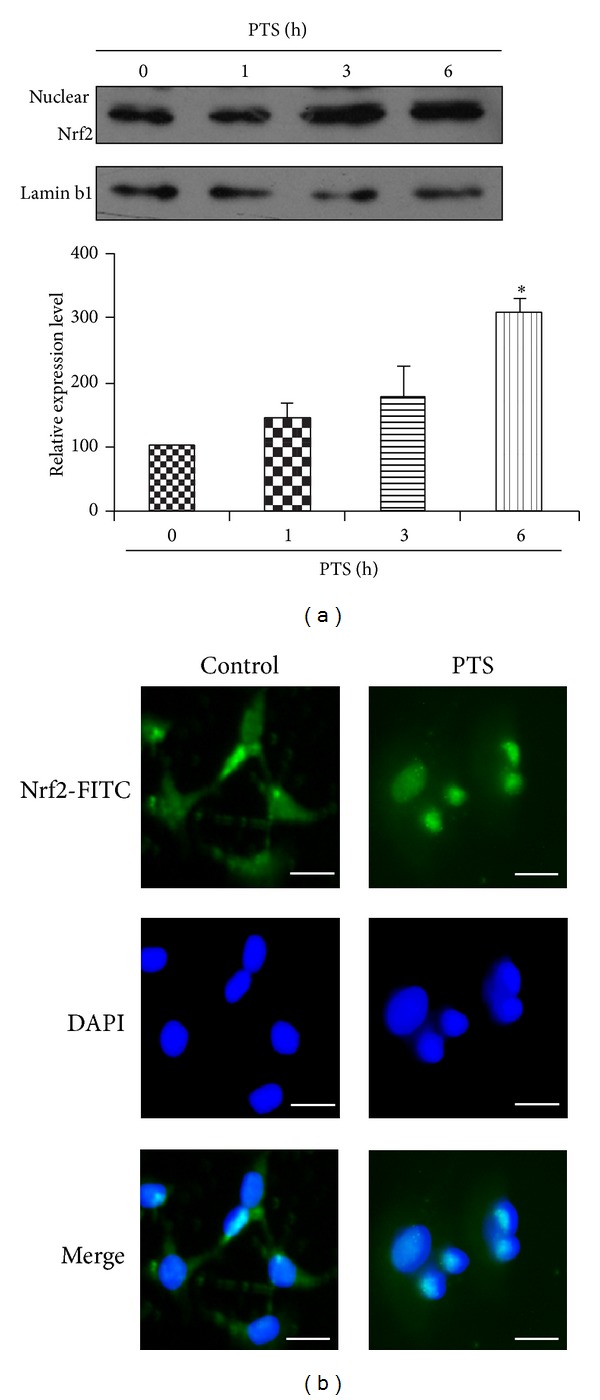
Effect of PTS on Nrf2 nuclear translocation in PC12 cells. (a) Determination of Nrf2 in the cell nucleus. PC12 cells were treated with PTS at fixed concentration (16 *μ*g·mL^−1^) for different time points as indicated. The nuclear proteins were isolated as described in Section 2. Nrf2 and nuclear marker lamin b1 were detected by Western blotting using specific antibodies. The blots were representative of three independent experiments. (b) Immunohistochemical determination of Nrf2 nuclear translocation. Cells were treated with 16 *μ*g·mL^−1^ for 6 h; then Nrf2 protein and nuclei were stained as described in Section 2. Three independent experiments were formed and the representative data are shown here. *P < 0.05, significantly different from vehicle group. Scale of bar is 50 *μ*m.

To investigate the potential molecular mechanism leading to HO-1 induction, we initially examined the nuclear translocation of Nrf2 in response to PTS (Figure 4). Nuclear proteins were isolated and analyzed by Western blotting using anti-Nrf2 antibodies, whereas lamin b1 was detected as a nuclear marker. As shown in Figure 4(a), the accumulation of Nrf2 proteins in the nucleus presented an increasing trend over the PTS (16 *μ*g·mL^−1^) treatment time of 0 to 6 h. Especially, significant induction of Nrf2 nuclear translocation was observed at the time point of 6 h. Then, we further characterized the Nrf2 nuclear translocation by immunohistochemical staining after treatment of PC12 cells with 16 *μ*g·mL^−1^ PTS for 6 h. As shown in Figure 4(b), Nrf2 stained with FITC presented mostly in the cytoplasm of untreated cells, whereas cells treated with PTS resulted in the appearance of FITC signal mostly in the nucleus. The green fluorescent staining of Nrf2 overlapped with blue DAPI staining of nucleus, suggesting the nuclear localization.

**Figure 5:**
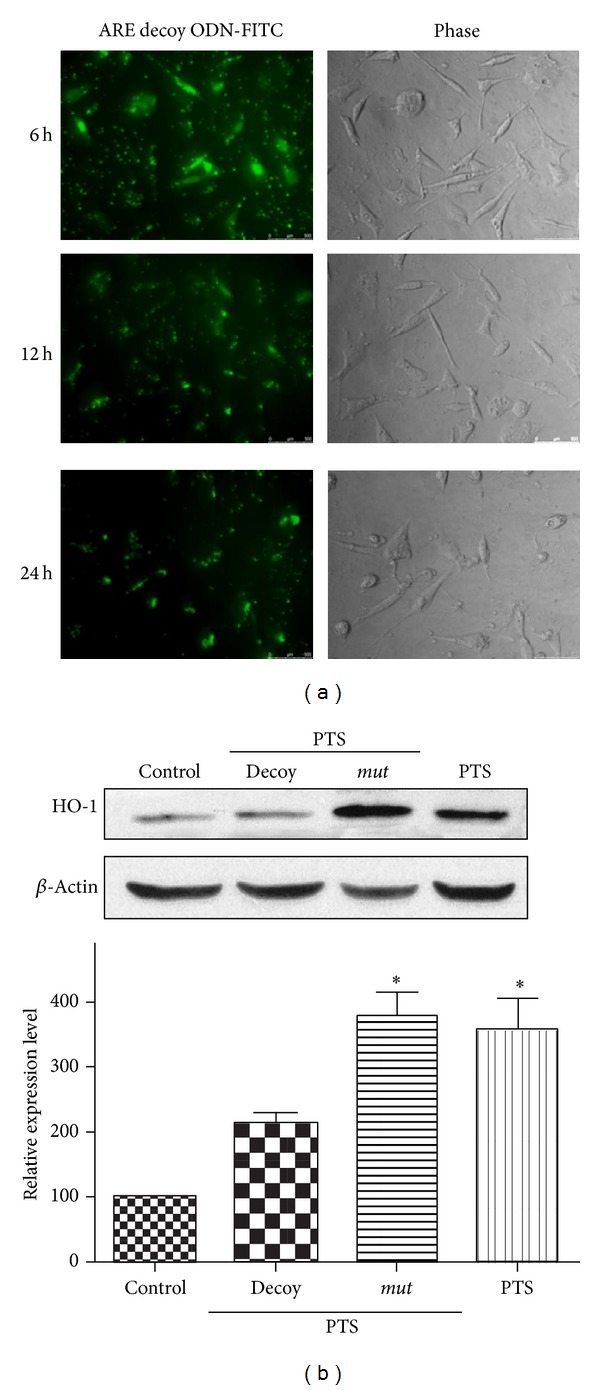
Effect of ARE decoy ODNs on PTS induced HO-1 expression in PC12 cells. (a) Cellular distribution of ARE decoy ODN-FITC. PC12 cells were treated with FITC-conjugated ARE decoy ODNs for 6 h, 12 h, and 24 h, respectively. Transfection and immunohistochemical determination were operated as described in Section 2. Scale of bar is 50 *μ*m. (b) Effect of ARE decoy ODNs on HO-1 induction by PTS. PC12 cells were directly exposed to 16 *μ*g·mL^−1^ PTS for 24 h, or following the treatment of ARE decoy or* mut* ODNs, protein was detected by Western blotting using specific antibodies against rabbit HO-1 and *β*-actin. Relative protein expression level of HO-1 was normalized to that of untreated cells after correction for the expression level of *β*-actin. The data were reported as mean ± SD (*n* = 3). *P < 0.05, significantly different from vehicle group. The blots were representative of three independent experiments.

To further prove that HO-1 induction by PTS treatment was directly regulated by Nrf2, we applied the decoy ODN strategy. In the decoy ODNs distribution experiment, FITC-labeled ARE decoy ODNs were administrated to PC12 cells and observed at different time points. Within 6 h of treatment with ARE decoy ODNs, a strong fluorescence labeling was observed in both cytoplasm and nucleus (Figure 5(a)). In addition, a part of fluorescence labeling was also observed in the extracellular space. Twenty-four hours after the treatment, the nuclear fluorescence had become much more intense, and the extracellular fluorescence had largely disappeared. As shown in Figure 5(b), HO-1 protein was significantly induced in PC12 cells after exposure to 16 *μ*g·mL^−1^ PTS for 24 h (*P* < 0.05), whereas no significant induction was observed after pretreatment of ARE decoy ODNs (*P* > 0.05). The pretreatment of a scrambled ARE* mut *ODNs did not influence the inducing ability of PTS on HO-1. The result indicated that induction of HO-1 by PTS was regulated by Nrf2 nuclear translocation and subsequently ARE binding.

### 3.3. PI3K/Akt Pathway Mediates Nrf2 Activation and HO-1 Induction by PTS

**Figure 6:**
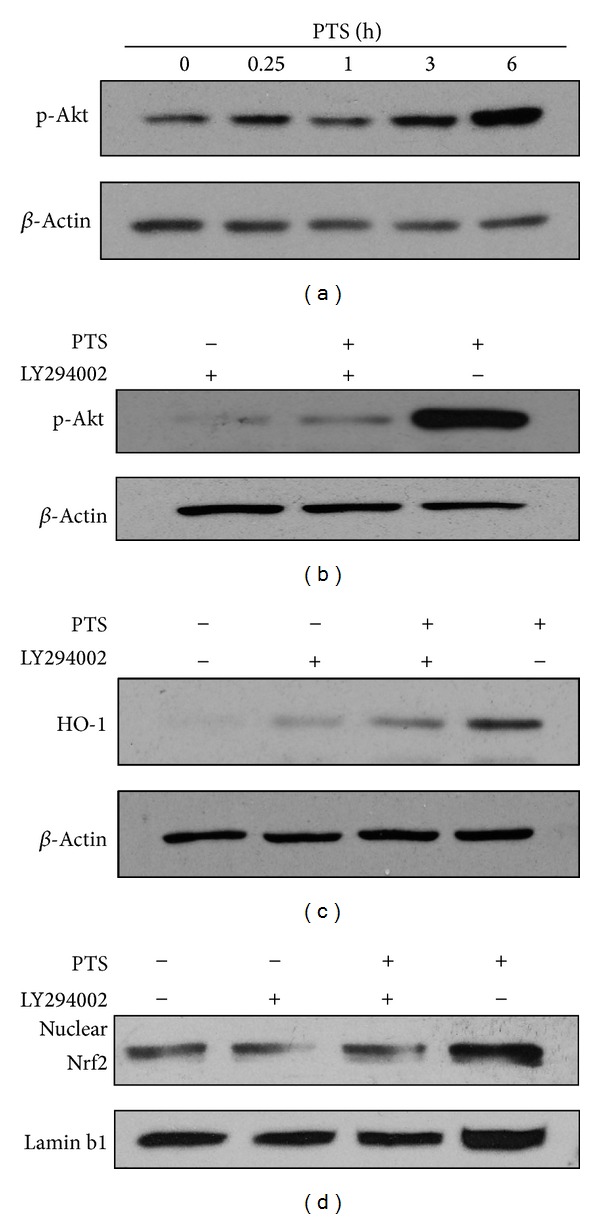
Role of PI3K/Akt in induction of HO-1 expression by PTS. (a) Phosphorylation of Akt induced by PTS. Cells were treated with PTS (16 *μ*g·mL^−1^) for different time points as indicated. (b) Inhibition of PI3K in PTS induced phosphorylation of Akt. Cells were treated with PTS (16 *μ*g·mL^−1^) in the absence and presence of 20 *μ*M LY294002 as indicated for 6 h. (c) Inhibition of PI3K in PTS induced HO-1 expression. Cells were incubated with PTS (16 *μ*g·mL^−1^) in the absence and presence of 20 *μ*M LY294002 for 6 h. (d) Inhibition of PI3K in PTS induced Nrf2 nuclear translocation in PC12 cells. Cells were treated with PTS (16 *μ*g·mL^−1^) in the absence and presence of 20 *μ*M LY294002 as indicated for 6 h. The blots were representative of three independent experiments.

To identify the potential upstream protein kinases involved in Nrf2 signaling pathway activation and HO-1 induction by PTS, the phosphorylation of Akt kinase induced by PTS was determined by Western blotting. The Akt phosphorylation was induced by PTS in a time-dependent manner (Figure 6(a)), suggesting that PI3 K/Akt kinase may play a role in PTS-stimulated Nrf2 activation. To further confirm this deduction, LY294002, an inhibitor specifically blocking PI3K/Akt signaling, was used. Akt phosphorylation, HO-1 expression, and Nrf2 nuclear translocation induced by PTS were effectively inhibited in the presence of LY294002 (Figures 6(b), 6(c), and 6(d)). These results suggested that PI3K/Akt kinase played an important role in PTS induced Nrf2 activation and HO-1 expression in PC12 cells.

### 3.4. Increased Induction of Phase II Enzymes by Combination of Rg1, Re, and R1 in PC12 Cells

**Figure 7:**
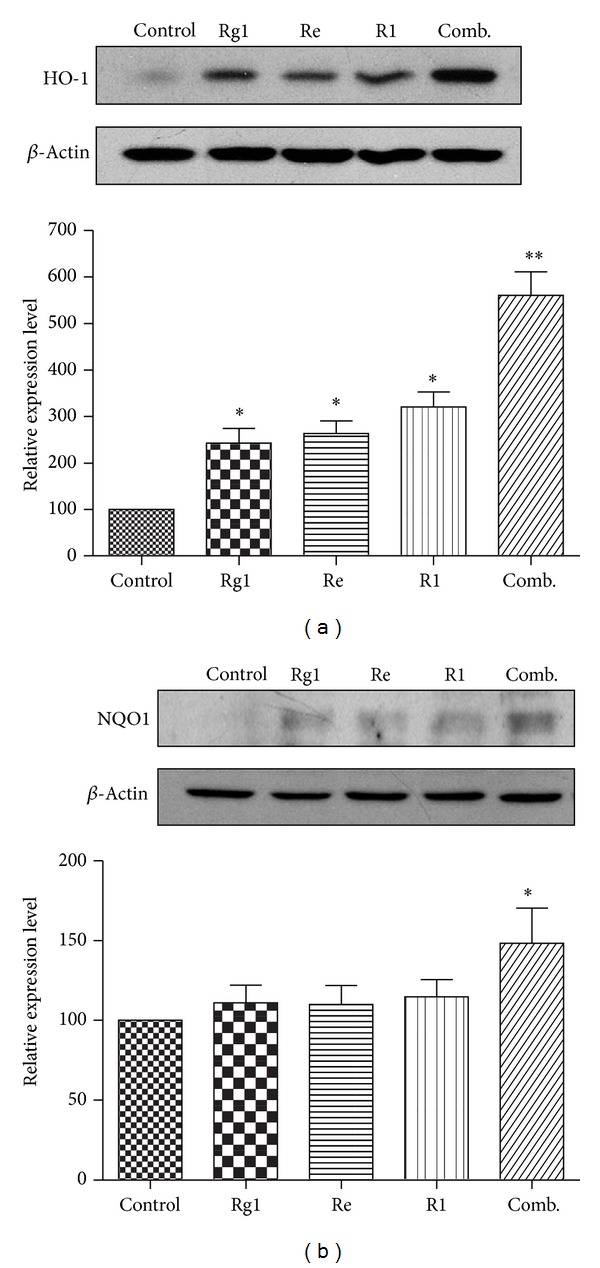
Increased induction of phase II enzymes by Rg1, Re, and R1 and their combination (Comb.) in PC12 cells. (a) Induction of HO-1. PC12 cells were treated for 24 h by Rg1 (2.5 *μ*M), Re (2.5 *μ*M), R1 (2.5 *μ*M), and their combination, respectively. Protein was detected by Western blotting using specific antibodies against rat HO-1, whereas *β*-actin protein was detected in parallel as the control. (b) Induction of NQO1. Following the treatment as described in Panel (a), protein was detected by Western blotting using specific antibodies against rat NQO1 and *β*-actin. Relative protein expression level of HO-1 and NQO1 was normalized to that of untreated cells after correction for the expression level of *β*-actin. The data were reported as mean ± SD (*n* = 3). *P < 0.05, **P < 0.01, significantly different from vehicle group. The blots were representative of three independent experiments.

The activation of Nrf2 signaling pathway induces a series of phase II enzymes, such as HO-1 and NQO1 [7], to protect cells against oxidative injury. To study the inducing effect on phase II enzyme by the combination of PTS main components Rg1, Re, and R1, we compared the induction of HO-1 and NQO1 at the protein levels by the three components and their combination. Both HO-1 and NQO1 levels were increased significantly after treatment with the combination of Rg1, Re, and R1 for 24 h (Figure 7). A 6-fold increase of HO-1 expression was made by the combination (*P* < 0.01), while a slight increase was made by each single compound (*P* < 0.05) (Figure 7(a)). A significant 1.5-fold of induction of NQO1 was observed by the combination (*P* < 0.05). Interestingly, none of the single compounds showed significant induction of NQO1 (*P* > 0.05) (Figure 7(b)). The results suggested that the combination of the three components significantly enhanced the induction of phase II enzymes in PC12 cells.

### 3.5. PTS Ameliorates OGD-Rep Induced Injury via PI3K/Akt and Nrf2 Signaling Pathway

**Figure 8:**
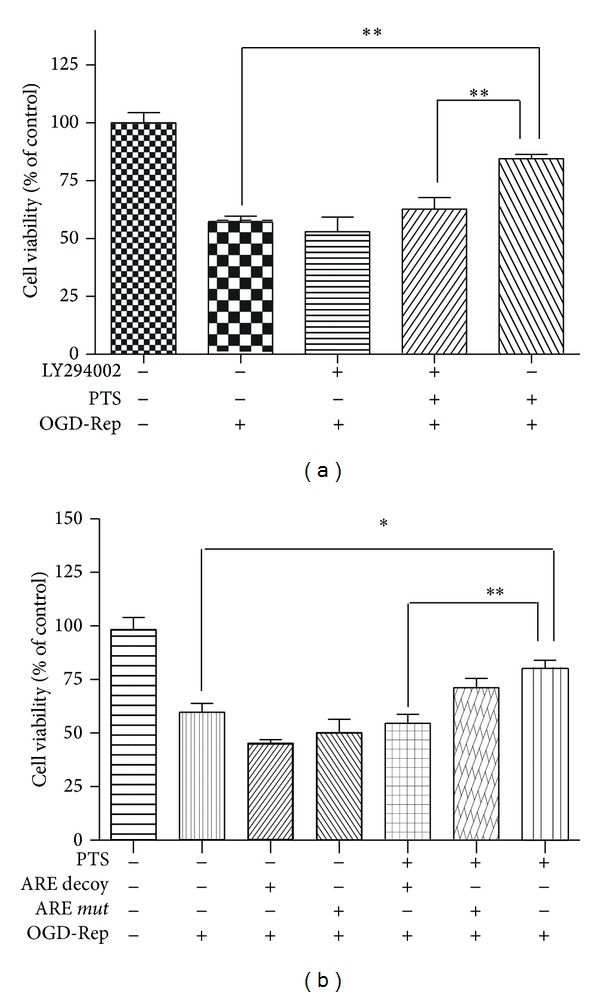
Role ofPI3K/Akt and Nrf2-Keap1 pathway in the protective effect of PTS against OGD-Rep. (a) PI3K inhibition on the protective effect of PTS against OGD-Rep. PC12 cells were treated with PTS (16 *μ*g·mL^−1^) in the absence and presence of 20 *μ*M LY294002 as indicated for 3 h, subsequently subjected to OGD-Rep treatment for 8 h, and finally maintained in oxygenated cell culture medium for another 24 h. The cell viability was determined by CCK-8 assay. (b) ARE decoy ODNs on the protective effect of PTS against OGD-Rep. Cells were directly exposed to PTS or treated with ARE decoy or* mut* ODNs, followed by PTS (16 *μ*g·mL^−1^) treatment for 3 h. Subsequently, the cells were subjected to OGD-Rep treatment and cell viability determination as indicated above. Values represent mean ± SD (*n* = 6). *P < 0.05 and **P < 0.01.

We have confirmed that PTS induced phase II enzymes through PI3K/Akt and Nrf2 pathway (Figure 8). To further determine the role of antioxidant phase II enzymes induced by PTS in its protective effect against OGD-Rep induced injury, we tested the effect of PTS again in the* in vitro* OGD-Rep cell model in the presence and absence of PI3K inhibitor or ARE decoy ODNs. As shown in Figure 7(a), PTS alone significantly protected PC12 cells against OGD-Rep induced cell death (*P* < 0.01), whereas this protective effect disappeared in the presence of 20 *μ*M LY294002. As determined in Figure 5(b), ARE decoy ODNs could inhibit the expression of Nrf2 driving phase II enzyme HO-1. In this study, ARE decoy ODNs eliminated PTS-mediated protection against OGD-Rep (*P* < 0.01) (Figure 7(b)). ARE* mut* ODNs treatment did not exhibit the same effect. It is thus clear that both LY294002 and ARE decoy ODN significantly decreased PTS-mediated protection against OGD-Rep, suggesting that PTS ameliorated OGD-Rep induced injury via PI3K/Akt and Nrf2 signaling pathway.

## 4. Discussion

Brain is deemed highly susceptible to oxidative stress due to a large amount of the body oxygen, a relatively poor antioxidant defense system, and a high concentration of prooxidant molecules [19]. ROS is readily generated during cerebral ischemia and reperfusion. Consequently, oxidative stress is easily caused and leads to neuronal cell death and brain damage, which is widely regarded as a key factor in the pathogenesis of ischemic stroke [4, 5]. The transcription factor Nrf2 is responsible for regulating a battery of antioxidant and cytoprotective genes, primarily in response to oxidative stress [6]. Therefore, it has received particular attention as a molecular target for pharmacological intervention of ischemic diseases [8]. Herbal medicines used in TCM and other folk medicines are now known to be rich in antioxidant and cytoprotective reagents [20]. By investigating novel neuroprotectants from TCM, we previously demonstrated that senkyunolide-H, -I [15] and Z-ligustilide [14] were antioxidant and cytoprotective via the activation of Nrf2-HO-1 signaling pathway.

PTS with Rg1, R1, and Re as three major compounds is one of the effective components of* Panax notoginseng*, which is beneficial to the prevention and treatment of cardio- and cerebrovascular diseases in China [10]. Previous animal study showed that pretreatment with PTS ameliorated ischemia reperfusion induced cerebral and myocardial damage [11, 12]. However, the role of Nrf2 signaling pathway in these processes is still not understood. Several ginsenosides (Rb1 [21], Rg3 [22], and Rg1 [23]) showed the ability to activate Nrf2 signaling pathway under different pathological insults in the previous study, which also gives us an impetus to perform this investigation.

The present study was designed to evaluate the effect of PTS on activating Nrf2 signaling pathway and the potential role in the protection against OGD-induced injury. In our* in vitro* ischemic stroke model, PTS at concentrations of up to 8 *μ*g·mL^−1^ significantly protected PC12 cells from oxidative damage. As expected, PTS also induced the expression of HO-1 protein in a concentration- and time-dependent manner. Further Western blotting and immunocytochemical staining demonstrated that PTS promoted the nuclear translocation of Nrf2. However, in addition to transcription factor Nrf2, HO-1 may also be turned on by several other transcription factors [24]. Thus, a transcription factor decoy approach was applied in our study. Sequence-specific inhibition of Nrf2 can be accomplished with synthetic double-stranded phosphorothioate oligonucleotides containing an Nrf2 consensus sequence, which acts as a “decoy”* cis*-element to bind transcription factors and block the activation of cognate genes [17, 25]. Our results showed that the ARE decoy ODNs brought about a marked decrease in the PTS induced HO-1 protein expression, whereas the control* mut* ODNs had no effect. These results demonstrated that the ARE decoy ODNs effectively competed with the cellular* cis*-element for binding the Nrf2 and interfered with the function of Nrf2 in intact cells, which also indicates that induction of HO-1 expression by PTS was directly regulated by Nrf2 activation.

It is reported that translocation of Nrf2 to the nucleus can be triggered by phosphorylation on serine 40. Nrf2 phosphorylation may be regulated through several signal transduction pathways, such as PI3K/Akt and mitogen-activated protein kinases (MAPKs) [26]. In this study, we first determined the potential effect of PTS on the phosphorylation of Akt and MAPK kinases (ERK, JNK, and p38). As a result, PTS had no effect on the phosphorylation of three MAPK kinases (data not shown). However, phosphorylation of Akt was significantly influenced by PTS. Inhibition of PI3K activity by specific inhibitor, LY294002, attenuated PTS induced phosphorylation of Akt, HO-1 expression, and accumulation of Nrf2 in the nucleus. These results indicated that PI3K may interact with the Nrf2 in the induction of downstream HO-1 by PTS. Usually, Akt is rapidly phosphorylated by activator. In our study, we found that phosphorylated Akt was significantly increased as early as 0.25 h. Then, it reached a maximum at 6 h. The long inducing duration was also reported previously. In HeLa cells, the phosphorylation of Akt was induced by etoposide within 6 h, while the maximum phosphorylation was observed at 3 h [27]. In H9c2 cardiomyocytes, p-Akt was induced with a highest phosphorylation by hypoxia-reoxygenation at 12 h [28]. Another reason for this long inducing duration of p-Akt by PTS might be its chemical complexity. However, the exact mechanism needs further study.

A recent study demonstrated that ginsenosides Rb1, Rg1, and 20S exhibited a synergistic manner in transcriptionally activating ARE [29]. It would be interesting to investigate the potential effect of the combination of Rg1, R1, and Re, the major compounds in PTS, on activation of Nrf2 signaling pathway. In our preliminary experiment, we have determined the inducing effect on HO-1 expression of the combination of Rg1, R1, and Re with the ratio presenting in PTS. However, the result indicated there was no increase in HO-1 induction with this combination (data not shown). However, the combination with equal concentrations of these three compounds exhibited significant stronger inducing ability for HO-1 and NQO1 than for each compound. But it is difficult to conclude that there is a synergistic effect at this stage.

Previous study demonstrated that Nrf2 signaling pathway may enhance the cellular resistance to ischemia-reperfusion induced oxidative stress via mediating downstream antioxidant proteins [8, 14]. Thus, we further determined the role of Nrf2 signaling pathway in the protective effect of PTS against OGD-Rep induced injury. The results showed that inhibition of PI3K activity by LY294002 significantly attenuated the protection of PTS. It is possible that inhibition of PI3K activity resulted in the inactivation of Nrf2 downstream regulating effect. Moreover, the PI3K/Akt pathway is also a key survival promoting signaling pathway [30, 31]. Therefore, it may also activate survival promoting signals and inhibit OGD-Rep induced cell death via the activation of Akt kinase in parallel. Meanwhile, we pretreated PC12 cells with ARE decoy ODNs to directly examine the role of Nrf2 signaling pathway in the protective effect of PTS against OGD-Rep induced injury. After inhibition of Nrf2-driven proteins with this decoy ODNs, the protective effect of PTS significantly reduced. The* mut* ODN did not show the same effect. The results demonstrated that Nrf2 regulated antioxidant and cytoprotective proteins may play a critical role in this protective process of PTS against OGD-Rep induced injury.

## 5. Conclusion

The present study examined the potential effect of PTS on the activation of Nrf2 signaling pathway and also the role of this cytoprotective pathway in PTS-mediated protection against OGD-induced injury. We found that PTS induced HO-1 expression and promoted Nrf2 nuclear translocation. With ARE decoy ODN strategy, we further confirmed that HO-1 induction by PTS was due to the direct activation of Nrf2 signaling pathway. Furthermore, PI3K/Akt kinase may also be involved in the upstream of Nrf2 signaling pathway by PTS. Moreover, the combination with equal concentration of Rg1, R1, and Re significantly increased the expression of phase II enzymes compared with each single compound. Finally, we proved that PI3K/Akt and Nrf2 signaling pathway were essential to enhance the cellular resistance to OGD-Rep induced cytotoxicity. In summary, our results suggest that PTS may activate the endogenous cytoprotective mechanism against OGD-Rep induced oxidative injury via the activation of PI3K/Akt and Nrf2 signaling pathway.

## Figures and Tables

**Table 1:** Sequence of decoy ODNs.

Name	Sequence
ARE decoy ODN	5′-CTAATGGTGACAAAGCAACTTT-3′
	3′-GATTACCACTGTTTCGTTGAAA-5′
ARE *mut* ODN	5′-CGACTGCCTTCAAAATAACTTT-3′
	3′-GCTGACGGAAGTTTTATTGAAA-5′
FITC-ARE decoy ODN	5′-(FITC)CTAATGGTGACAAAGCAACTTT-3′
	3′-GATTACCACTGTTTCGTTGAAA(FITC)-5′
